# Modified Sequential Therapy Regimen versus Conventional Triple Therapy for *Helicobacter Pylori* Eradication in Duodenal Ulcer Patients in China: A Multicenter Clinical Comparative Study

**DOI:** 10.1155/2012/405425

**Published:** 2011-11-21

**Authors:** Ying-Qun Zhou, Ling Xu, Bing-Fang Wang, Xiao-Ming Fan, Jian-Ye Wu, Chun-Yan Wang, Chuan-Yong Guo, Xuan-Fu Xu

**Affiliations:** ^1^Department of Gastroenterology, Shanghai Tenth People's Hospital, Tongji University, Shanghai 200072, China; ^2^Department of Gastroenterology, Kunshan Hospital, Jiangsu University, Jiangsu Province, Kunshan 215300, China; ^3^Department of Gastroenterology, Jinshan Hospital, Fudan University, Shanghai 200540, China; ^4^Department of Gastroenterology, Third People's Hospital of Changzhou, Jiangsu Province, Changzhou 213001, China; ^5^Department of Gastroenterology, People's Hospital of Hangzhou Under District, Zhejiang Province, Hangzhou 310003, China

## Abstract

*Objective*. Antimicrobial resistance has decreased eradication rates for *Helicobacter pylori* infection worldwide. To observe the effect of eradicating *Helicobacter pylori (H. pylori)* and the treatment of duodenal ulcer by 2 kinds of modified sequential therapy through comparing with that of 10-day standard triple therapy. *Methods*. A total of 210 patients who were confirmed in duodenal ulcer active or heal period by gastroscopy and *H. pylori* positive confirmed by rapid urease test, serum anti-*H. pylori* antibody (ELASE), or histological examination enrolled in the study. All the patients were randomly divided into three groups: group A (70 cases) and group B (70 cases) were provided 10-day modified sequential therapy; group C (70 cases) was provided 10-day standard triple therapy. Patients of group A received 20 mg of Esomeprazole, 500 mg of Clarithromycin for the first 5 days, followed by 20 mg of Esomeprazole, 500 mg of Clarithromycin, and 1000 mg of Amoxicillin for the remaining 5 days. Group B received 20 mg of Esomeprazole, 1000 mg of Amoxicillin for the first 5 days, followed by 20 mg of Esomeprazole, 500 mg of Clarithromycin, and 1000 mg of Amoxicillin for the remaining 5 days. Group C received 20 mg of Esomeprazole, 500 mg of Clarithromycin, and 1000 mg of Amoxicillin for standard 10-day therapy. All drugs were given twice daily. *H. pylori* eradication rate was checked four to eight weeks after taking the medicine by using a ^13^C urea breath test. In the first, second, third, seventh, twenty-first, thirty-fifth days respectively, the symptoms of patients such as epigastric gnawing, burning pain, and acidity were evaluated simultaneously. *Results*. Overall, 210 patients accomplished all therapy schemes, 9 case patients were excluded. The examination result indicated that the *H. pylori* eradication rate of each group was as follows: group A 92.5% (62/67), group B 86.8% (59/68), and group C 78.8% (52/66). The *H. pylori* eradication rate of group A was slightly higher than group B (*P* < 0.05) and both of them were obviously higher than group C (*P* < 0.05). Modified sequential therapy was significantly more effective in patients with clarithromycin-resistant strains (80%/67% versus 31%; *P* = 0.02). Symptoms improvement: all the three groups could improve the symptoms such as epigastric gnawing, burning pain, and acidity since the first day. There was no significant difference in total score descending of symptoms between each group (*P* > 0.05). *Conclusions*. All the three therapy schemes could alleviate symptoms of duodenal ulcer patients in China efficiently. But as far as eradicating *H. pylori* is concerned, the modified sequential therapy was better than standard triple therapy, especially the therapy scheme used in group A.

## 1. Introduction

*Helicobacter pylori* (*H. pylori, Hp*) plays a crucial role in the pathogenesis of chronic gastritis, peptic ulcer disease (gastric ulcer, duodenal ulcer), gastric-mucosa-associated lymphoid tissue (MALT) lymphoma, and gastric adenocarcinoma. *H. pylori* is a major cause of illness and death worldwide [1]. The treatment of duodenal ulcer mainly depended on *H. pylori* eradication and proton pump inhibitor (PPI). Obvious effect was also achieved. *Helicobacter pylori* treatment still remains a challenge for physicians, and no current first-line therapies are able to cure the infection in all treated patients [2]. The standard *H. pylori* eradication therapy in China consists of a triple-drug regimen containing a proton pump inhibitor (bismuth) and clarithromycin, with either amoxicillin or an imidazole [3]. Indeed, during the last few years, different studies have found that the success rate following such regimens is disappointingly low, with values less than 45–60% in some countries [4]. This phenomenon most likely depends on an increased bacterial resistance to antibiotics, particularly against clarithromycin the key antibiotic in *H. pylori* treatment [5]. However, it has also been concerned about the eradication rates with those regimens decreased due to emergence of metronidazole resistance in *H. pylori* over the past few years. GUO et al. [6] have reported that prevalence of metronidazole-resistant *H. pylori* strains has increased to more than 70% in China. Two large meta-analyses [7, 8] showed that these therapies failed to eradicate *H. pylori *in up to 20% of patients. Even lower cure rates have been observed in primary medical care settings, with bacterial eradication being achieved in only 61% to 76% of patients. So we need new treatment regimens.

De Francesco et al. [9] discovered a new program to eradicate *H. pylori* sequential therapy, which can obtain a higher eradication rate, but related reports in China are still not abundant. Novel 10-day sequential therapy consisting of 5-day dual therapy (proton pump inhibitor plus amoxicillin) followed by 5-day triple therapy (proton pump inhibitor, Clarithromycin, and tinidazole) had good eradication success in unblinded trials in elderly patients [10, 11]. This study compared two kinds of modified sequential therapy and a kind of conventional triple therapy to explore the difference in eradication of *H. pylori* and treatment of duodenal ulcer and such improvement of symptoms. Our study achieved some meaningful results as follows.

## 2. Materials and Methods

### 2.1. General Data of Patients

This was a comparative, parallel, open-label, multicenter, randomized study. It was carried out between January 2007 and July 2009.

Criteria of inclusion: (1) age 15–70 years old, no restrict gender, (2) duodenal ulcer inactive and heal period confirmed by gastroscopy in seven days, (3) intensive positive in rapid urease test (RUT) and *H. pylori (Hp)*-positive confirmed by serum anti-*Hp* antibody, stained slice, or histological examination, and (4) approve to participate the efficiency observation.

Criteria of exclusion: (1) patients who had gastric ulcer or severe gastroesophageal reflux disease must take proton pump inhibitor (PPI), patients who had gastrectomy history, patients who had hemolytic anemia or family history, (2) patients who were in lactation or pregnancy, (3) patients who had combined severe disease of other system that may affect the medical evaluation of this study, (4) patients who took the drugs of this study in one month, (5) patients who was allergic to the drugs in this study, and (6) patients who had dysfunction of heart, liver, and kidney.

Criteria of suspension: (1) patients who had poor compliance and could not take medicine at arrangement, (2) patients who combined application of other treatment such as acid inhibitor and antiacid medicine, and (3) patients who were considered should not take the medicine by doctor according to their benefit because adverse accident happened. These results of case patients did not participate in effect statistic but in safety evaluation, and (4) patients who could not reexam in time or lost. All participants gave written informed consent.

### 2.2. Therapy Regimens

In each center, all patients were randomly assigned using a computer-generated list to one of the following treatments. Group A and group B were provided 10-day modified sequential therapy. Group C was provided 10-day standard triple therapy. Patients of group A received 20 mg of Esomeprazole, 500 mg of Clarithromycin for the first 5 days, followed by 20 mg of Esomeprazole, 500 mg of Clarithromycin, and 1000 mg of Amoxicillin for the remaining 5 days. Group B received 20 mg of Esomeprazole, 1000 mg of Amoxicillin for the first 5 days, followed by 20 mg of Esomeprazole, 500 mg of Clarithromycin, and 1000 mg of Amoxicillin for the remaining 5 days. Group C received 20 mg of Esomeprazole, 500 mg of Clarithromycin, and 1000 mg of Amoxicillin for standard 10-day therapy. All drugs were given twice daily. Esomeprazole (40 mg/tab, 010672, pharmaceutical Co. Ltd), clarithromycin (250 mg/tab, 030501, Hangzhou Chinese-American East China Pharmaceutical Co. Ltd), and Amoxicillin (500 mg/tab, 011003, Pharmaceutical factory).

 For each therapy regimen, the proton pump inhibitor was prescribed before breakfast and supper, whereas all antibiotics were given after these meals. Patients were asked to return at the end of the antibiotic treatment to assess the compliance with therapy and to estimate the incidence of side effects. Compliance was defined as consumption of greater than 90% of the prescribed drugs and was determined by pill counts at the followup visit. Side effects were evaluated using a structured questionnaire by personal interview.

### 2.3. Items Observed and Examined

**Figure 1:**
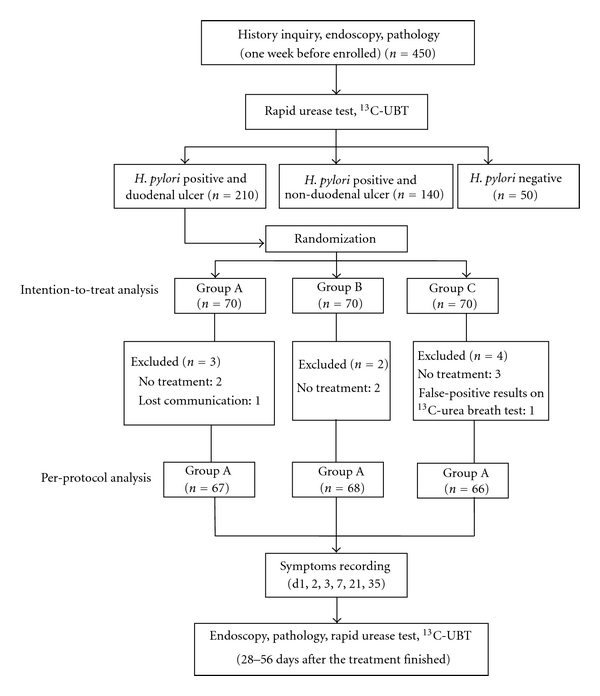
Study regimen.

Illness history clinical symptom was recorded and gastroscopy was executed to clarify the disease diagnosis and status of *H. pylori* infection. During the gastroscopy examination, four biopsy specimens were taken from gastric antrum: one for RUT, one for stained slice, and two additional biopsy samples for bacterial culture and susceptibility testing. For this purpose, biopsy specimens were sent to a single microbiological laboratory in Shanghai within 24 hours and were stored at −70°C. Isolated strains were considered to be resistant to clarithromycin and amoxicillin if minimal inhibitory concentrations were ≥1 ug/mL and ≥0.8 ug/mL, respectively. Serum anti-*Hp* antibody was also probed (ELASE). The patients who were intensive positive in RUT (show red in five minutes) could be considered qualified. Those who were suspected to be *H. pylori* positive would take additional examinations by silver dying and improved Giemsa dying. Besides RUT positive, all participants must be positive in anti-*Hp* antibody, *H. pylori* dying, and histological examination. In d1, 2, 3, 7, 21, and 35, the symptoms of patients such as epigastralgia, burning pain, and acidity were evaluated; the adverse reaction and the compliance of the patients were also assessed. Bacterial eradication was checked 28 to 56 days after the treatment finished by using a ^13^C-urea breath test.  Figure 1 shows the flow of patients through the study. The other outcome was to determine the efficacy of sequential treatment against clarithromycin-resistant strains of *H. pylori*.

### 2.4. Symptoms Score and Effect Evaluation

The symptoms during the first day and at the end of 2, 3, 7, 21, and 35 days were monitored by scoring symptoms. Symptoms score, included the symptoms of epigastralgia, burning pain, and acidity. Score criteria: score 0 for no symptoms; score 1 for slight, symptoms could be remarked; score 2 for medium, had chief complaint of symptoms but did not affect daily life; score 3 for severe, had symptoms and affected daily life. Each patient was taught how to evaluate and make a record on a diary card. Criteria of total effect evaluation [12, 13]: obvious effect for the rate of score descending higher than 75%; effect for the rate higher than 50% but lower than 75%; progress for the rate higher than 25% but lower than 50%; no effect for the rate lower than 25%. Symptoms improvement evaluation: disappearance rate of chief symptoms was the rate of chief symptoms disappearing entirely; alleviation rate was the rate of symptoms improvement including obvious effect, effect, and progress. Safety evaluation: safety compare of different schemes treatment.

Endoscopic images before and after therapy were considered another indicator of effective treatment. For the purposes of this study, the duodenal ulcer was confirmed in active or heal period by gastroscopy in seven days. Endoscopists in each center received training before the trial started and two copies of pictures from every patient were sent to the leading center for repeat reviewing. Safety monitoring was also conducted according to the recorded adverse events.

### 2.5. Cost Calculations

The cost of each treatment was estimated by using retail US costs obtained from our study. The retail cost of the medications in China was as follows: 500 mg of amoxicillin, $0.16; 250 mg of clarithromycin, $0.75; 20 mg of Esomeprazole, $2.13. The calculated costs for 10-day modified sequential therapy and standard therapy were $75.6 (Group A), $63.8 (Group B), and $78.8 (Group C), respectively. The cost of sequential therapy was cheaper than standard triple therapy. Therefore, the modified sequential therapy is an economical alternative to standard triple therapy.

### 2.6. Statistical Analysis

The sample size was a priori calculated based on available data in the literature [14]. By hypothesizing a 95% eradication rate for the sequential regimen and 80% for 10-day triple therapy, it was calculated that at least 68 patients per treatment arm were needed to find a statistically significant difference with a level of *P* < 0.05 and a power of 0.85 [15]. *H. pylori* eradication rate was the main analytic target. Total eradication rate and its 95% confidence interval of each scheme were calculated and analyzed by ITT (intention to treat analysis) and PP (per-protocol analysis), respectively. The eradication rate difference of each scheme and degree of symptoms improvement were made significant check by Fisher exact probability and Chi-square test, the significant level was definite *P* < 0.05. The different significance of adverse reaction incidence rate of each scheme was analyzed by Fisher exact probability test.

## 3. Results

### 3.1. General Data

There were total 210 patients enrolled in the study, 201 patients accomplished actually (95.7%), and 9 patients were lost (4.3%). Details of groups division were as follows: group A, 67 patients, 40 patients male, 27 patients female, 15–69a, mean 38 ± 10a, 3 patients lost; group B, 68 patients, 43 patients male, 25 patients female, 17–70a, mean 36 ± 11a, 2 patients lost; group C, 66 patients, 40 patients male, 26 patients female, 18–69a, mean 37 ± 10a, 4 patients lost. The gender, age structure, and lost patients of the three groups had no significant distinction (*P* > 0.05) (Table 1).

### 3.2. Effect and Symptoms Improvement Analysis

**Figure 2:**
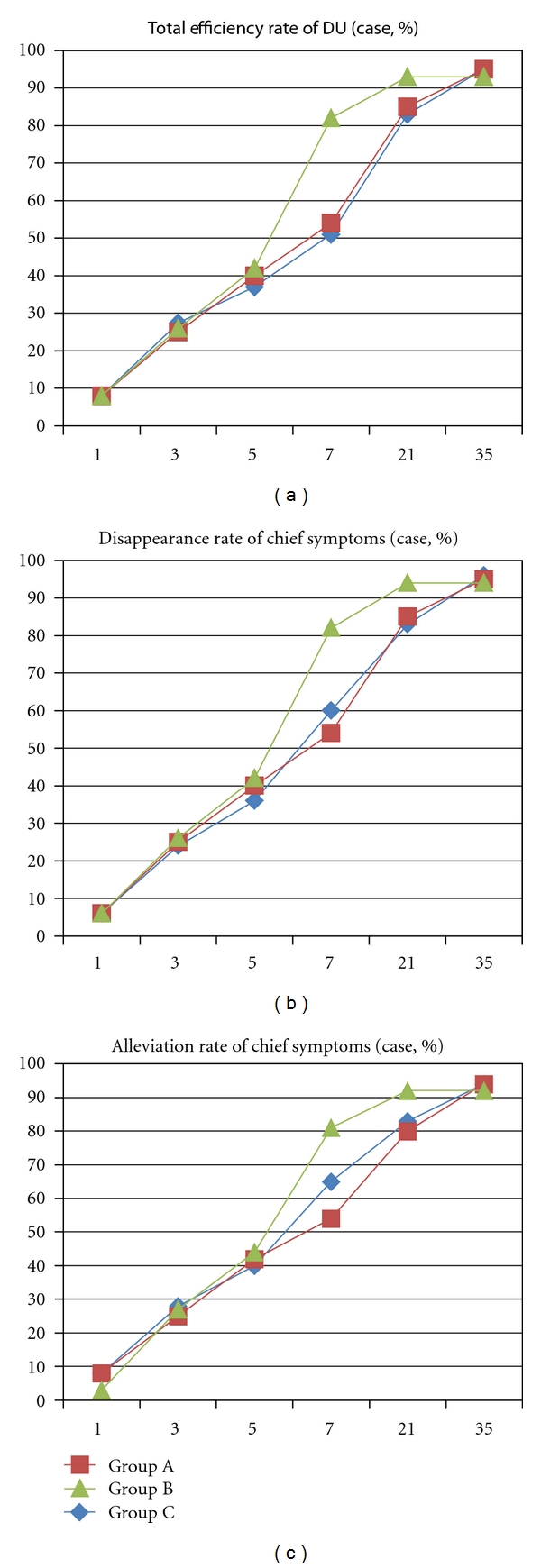
The trend of duodenal-ulcer-associated symptoms in the four groups. Time courses of the mean value of symptom scores are shown in the graphs. The *x*-axis represents days from baseline and the *y*-axis represents the change rate of symptom score in DU patients. (The lines represent the trend rather than a continuous variable).

All the three groups could improve the symptoms such as epigastralgia, burning pain and acidity since the first day. There was no significant distinction in total score descending of symptoms between each group (*P* > 0.05). There was no significant distinction in total efficiency rate of duodenal ulcer treatment, disappearance rate, and alleviation rate of chief symptoms of the three groups (*P* > 0.05) (Figure 2). 

### 3.3. *H. Pylori* Eradication Rate

The result of *H. pylori* eradication rate examination indicated that by per-protocol analysis (PP) the *H. pylori* eradication rate of group A was 92.5% (62/67), the rate of group B was 86.8% (59/68), the rate of group C was 78.8% (52/66), and by intention-to-treat analysis (ITT) the rate of group A was 88.6% (62/70), the rate of group B was 84.3% (59/70), and the rate of group C was 74.3% (52/70). The *H. pylori* eradication rate of group A was slightly higher than group B (*P* < 0.05), and both of them were obviously higher than group C (*P* < 0.05) (Table 2).

### 3.4. Role of Primary Resistance on Eradication of *Helicobacter Pylori* Infection

Bacterial culture was successful in 178 of 210 (85%) patients. Isolated clarithromycin resistance was present in 24 of 210 (13.5%) strains. No bacterial resistance to amoxicillin was observed. In sequential therapy, the prevalence of primary bacterial resistance to clarithromycin was 5 of 70 (7.1%) and 6 of 70 (8.6%) patients, respectively. In standard therapy, the prevalence of strains resistant to clarithromycin was 13 of 70 (18.6%) patients. The eradication rate was 80% and 67%, respectively in patients with isolated clarithromycin resistance who received sequential therapy compared with 31% of patients who received standard therapy. However, the difference was not statistically significant in patients with clarithromycin susceptible strains, receiving the modified sequential regimen comparing with those receiving standard treatment (89%/86% versus 84%, *P* = 0.71).

### 3.5. Adverse Reaction

Both treatments were well tolerated and no patient was withdrawn from the study. The most frequent adverse reaction related to the treatment was gastroenteric reaction. Of all the patients, group A had 8 cases, group B had 6 cases, and group C had 7 cases. But all the adverse reactions of each group were relatively slight. The compliance of patients was well (Table 3).

## 4. Discussion

*Helicobacter pylori *(*H. pylori*) is a common type of bacteria that usually infects people during childhood. In almost 50% of cases, the infection does not cause symptoms. However, some people with *H. pylori* infection eventually develop inflammation of the stomach (gastritis) or ulcers in the stomach or upper small intestine [5, 16–18]. Gastritis and ulcers cause abdominal pain and, sometimes, bleeding. *H. pylori* causes significant morbidity and mortality with a relevant economic impact, thus requiring an appropriate therapeutic approach. A triple therapy, comprising proton pump inhibitor, clarithromycin, and amoxycillin or metronidazole, is among the most used first-line treatments in primary medical care in several countries including Canada, the United States, and Europe [14, 15, 19–21]. Doctors often treat stomach pain and ulcers caused by *H. pylori *with a combination of several antibiotics that are given for several days. Some data confirm the finding of disappointing eradication rates after standard triple therapy, as widely reported in several recent studies [22–30]. Indeed, one of every six patients with peptic ulcer disease remained infected after standard therapy [29]. In recent years, there has been increasing resistance to standard antibiotic treatments for *H. pylori* infection [31–33]. This means that it is harder to get rid of *H. pylori* in some patients and that we need new treatment regimens.

Low eradication rates have been reported with standard therapy in the United States, Europe, Australia, and Asia [4, 34, 35]. Our study confirms these reports on the low success rates of infection eradication with standard therapy. This may be mainly due to clarithromycin resistance. After a decade of clarithromycin-based treatments and continued widespread use of long-acting macrolides in general practice, 10% to 15% of *H. pylori* strains are resistant de novo to clarithromycin [36]. As a result, the failure rate is around 20% for triple combination therapy (PPI plus amoxicillin plus clarithromycin), which was so effective when it was first evaluated 10 years ago [37, 38]. Because persistent *H. pylori* in patients with ulcer can cause continuing ulcer complications, a failure rate of 20% also means that everyone needs follow-up proof of cure. In addition, the 20% of patients with persistent *H. pylori* warrant repeated attempts at eradication with ever-decreasing success. A systematic review of therapy for* H. pylori* reported a 53% decrease in eradication rates if clarithromycin resistance was present and if a clarithromycin-containing regimen was used [39]. Clarithromycin resistance is a major problem in many western countries. Prevalence is 12.9% in the United States and may be as high as 24% in some European countries [40–42].

In contrast, we found a high *H. pylori* eradication rate using the sequential regimen. It was significantly greater than that of standard triple therapy. Moreover, the sequential treatment showed high efficacy (>90%) in patients with peptic ulcer disease and nonulcer dyspepsia [43]. Sequential therapy for *Helicobacter pylori* refers to the idea of adding more antibiotics to the treatment regimen but giving them in sequence rather than giving all 4 drugs together. In a large, prospective, controlled study in 2007, Vaira and colleagues [43] showed a 90% cure rate for this “new” treatment versus 80% for the “old.” In this issue, Jafri and colleagues [44] perform a meta-analysis of clinical trials of sequential therapy. Staggering the treatment with multiple antibiotics does not increase side effects but still eradicates almost all *H. pylori* isolates, the exceptions being doubly resistant isolates. Thus, sequential therapy combines the initial and the repeated therapy in 1 treatment sequence, for the same cost and with the same side effect profile as those of the present standard therapy.

The precise mechanism for the success of the sequential therapy is not known; however, bacteria can develop efflux channels for clarithromycin, which rapidly transfer the drug out of the bacteria cell, preventing the antibiotic from binding to the ribosome [45]. Because amoxicillin acts on the bacterial cell wall and weakens it, the initial phase of treatment may prevent the development of efflux channels by weakening the cell wall of the bacterium [45]. This may improve the efficacy of clarithromycin in the second phase of treatment. The higher efficacy of the sequential regimen may be related to the larger number of antibiotics (3 drugs) to which the organism is exposed with this regimen or to the use of tinidazole, which is not contained in the standard triple-drug regimen. Sequential therapies are relatively new, and few data on these regimens are in the literature [43]. A MEDLINE search to May 2011 by using the keywords “sequential therapy” and “*Helicobacter pylori*” yielded 168 citations. Recently Uygun A and colleagues [46] perform a study to compare the eradication success of a 14-day sequential regimen with proton-pump-inhibitor-based triple treatment. They found that a 14-day sequential treatment regimen achieved a significantly higher eradication rate of *H. pylori* compared with standard PPI-based triple regimen in this selected population. Hsu and colleagues [47] showed that there was a multicenter pilot study in which *H. pylori*-infected patients received a 14-day sequential-concomitant hybrid therapy (esomeprazole and amoxicillin for 7 days followed by esomeprazole, amoxicillin clarithromycin, and metronidazole for 7 days). The eradication rate was 99.1% (95% confidence interval (CI), 97.3–100.0%) by per-protocol analysis and 97.4% by intention-to-treat analysis (95% CI, 94.5–100.0%). Cost is a major consideration in many countries. The cost of the sequential regimen is similar to that of the standard regimen in China, which makes it an attractive and reasonable alternative to triple therapy [48–50].

Sequential therapy is currently suggested as first-line therapy in curing *Helicobacter pylori* infection, but results coming from its use in clinical practice are scarce [51]. We evaluated the efficacy of this therapy regimen in our current clinical practice. The results of this study show that modified sequential therapy is superior to triple therapy for the eradication of *H. pylori *infection. Our study also demonstrates that additional antibiotic can improve effect. Triple therapy, which is the current standard treatment, has low eradication rates. In conclusion, our large, prospective, controlled study shows the superiority of sequential treatment for eradicating *H. pylori* infection in duodenal ulcer patients compared with conventional triple therapy. All treatments were well tolerated and had similar rates of side effects and low rates of patients who withdrew from the study. Our study suggests that modified sequential therapy may have a role as a first-line treatment for *H. pylori *infection in duodenal ulcer patients in China. Modified sequential regimen seems to be a valid therapeutic strategy for the management of *H. pylori* infection in clinical practice.

##  Conflict of Interests

The authors have no competing interests to report. This paper represents original research that has not been published nor submitted for publication elsewhere.

## Figures and Tables

**Table 1:** Characteristics of patients entering the intention-to-treat analysis.

	Group A (*n* = 67)	Group B (*n* = 68)	Group C (*n* = 66)	*P* value
Age (mean ± SD, years)	38 ± 10	36 ± 11	37 ± 10	0.6
Sex				
Male	40	43	40	0.1
Female	27	25	26	0.1

SD: standard deviation.

**Table 2:** *Helicobacter pylori* eradication rates with sequential and standard therapy both at intention-to-treat (ITT) and per protocol (PP) analyses.

Analysis	Group A	Group B	Group C
ITT (%)*	62/70 (88.6%)	59/70 (84.3%)	52/70 (74.3%)
95% CI	80.3 to 92.9	79.2 to 93.2	63.5 to 78.8
PP (%)^†^	62/67 (92.5%)	59/68 (86.8%)	52/66 (78.8%)
95% CI	79.2 to 95.8	78.0 to 94.6	65.9 to 84.6

All patients who took at least 1 dose of medication are included. *Group A versus Group B therapy: *P* = 0.4; Group A versus Group C therapy: *P* = 0.03; Group B versus Group C therapy: *P* = 0.04; ^†^Group A versus Group B therapy: *P* = 0.02; Group A versus Group C therapy: *P* = 0.01; Group B versus Group C therapy: *P* = 0.02.

**Table 3:** Patients with self-reported adverse events during therapy.

Adverse Event (*n*)	Group A (*n* = 67)	Group B (*n* = 68)	Group C (*n* = 66)
Epigastric pain	4	3	5
Skin Rash	1	0	2
Headache	2	2	0
Glossitis	0	0	0
Anergy	0	0	0
Fever	0	1	0
drowsiness	1	0	0
Total, *n* (%)	8 (11.9)	6 (8.8)	7 (10.6)
